# War and Disease. I.—Continental Wars of the Nineteenth Century

**Published:** 1915-01-23

**Authors:** 


### WAR AND DISEASE.

### Continental Wars of the Nineteenth Century.

(Being the substance of the first of three Chadwick Trust lectures delivered by Col. F. M. Sandwith, M.D., Chairman of the British Red Cross Society, at the Royal Society of Arts on January 15.)

Dr. Sandwith said there could hardly be a man or woman in the great nations of Europe who had not, during the past five months, weighed many times the chances of success or failure in this great war. All the departments of activity had played their part, and the excellence or the inferiority of each had aided or detracted from the success of the plans worked out by the leaders and by our formidable enemy. And there was a general conviction that success must lie with those whose spirit endured, and whose *morale* remained high. But the medical officer realised that the sternest determination and the most inflexible courage were, to a certain extent, dependent upon physical conditions. Pain, hunger, cold, and sickness—any causes of lowered vitality—tended to reduce the soldier's physical and moral endurance, and these complications might do more harm than the enemy bullets. Our knowledge to-day was based upon sounder principles, and the mighty armies to-day were better equipped and instructed, and better fitted to endure hardships in a long and bitter struggle than in any previous campaign. There had been only one previous instance in which the soldier was fitted for his task—namely, in the case of the Japanese Army in the war with Russia in 1904-5. In Japan's previous war with China she lost three men from disease to each man from warfare.

### Early Days of the Army Medical Department.

In recent wars fought on land the average mortality worked out at twenty deaths from wounds and eighty from disease out of every 100; this was the proportion in the Russo-Turkish War of 1877-8. In the American Civil War there were three deaths from disease to one from wounds. In the French Expedition to Madagascar, in 1895, twenty-nine were killed in action to 7,000 dead from disease. In the United States Army during the Spanish-American War, fourteen died from disease to every one killed in action, though the war lasted little more than six weeks. In the Boer War the British losses from disease were terrible, in spite of considerable improvements in military hygiene. In the Russo-Japanese War, the Japanese, by being prepared for every eventuality, and paying attention to the smallest details concerning the health of the troops, were able to reverse the previously accepted proportion of four dead from disease to one dead from wounds, for only one died from disease to every four who died from wounds. This success was due to their adaptation of the best hygienic principles to the life of the soldier, in peace as well as in time of war; it carried out the principle that all knowledge should, primarily, be for the service of the State.

John Howard, of prison reform fame, and Sir Edwin Chadwick were stimulated in their reforming zeal by outbreaks of epidemics which frightened local authorities, and this led to the appointment of the first Sanitary Commission, of which Chadwick was a member; and though Sir Edwin retired from official life in 1854, his zeal in matters of hygiene never abated, and he was busy with inquiries as to the sanitary condition of our army, which was then suffering greatly in the Crimea, largely from preventable privations. William Howard Russell's letters to the *Times* aroused the public to the realisation of what the men were suffering. At one time there were no sick hospitals and no nurses at the seat of war, and even in the Napoleonic wars the care of the sick and wounded was lamentably neglected. Matters were but little better in the Peninsular War. In 1854 the Army Medical Department consisted of one Director-General, one assistant, and about six clerks.

### The Crimean Revelations.

Moreover at the outbreak of the Crimean War the Director-General struggled hopelessly to cope with requirements on grossly insufficient funds. The result of placing our soldiers in the hands of untrained persons was terrible: Cholera, dysentery, typhus fever, scurvy, frost-bite, and every condition which followed upon starvation decimated the ranks in the fighting line. In January 1855 there were 23,076 sick, seven-eighths being due to exposure and privation. At this time Sidney Herbert, a man of fearless and independent character, became Secretary of State for War, and, with the co-operation of Florence Nightingale, he got a staff of nurses at the Front, and drastic reforms were introduced in the spring of 1855. In response to the famous lady's request, a Commission was sent from home to the Crimea, and resulted in the construction of hospitals and the improvement of the water supply, and this step, according to Florence Nightingale, saved the British Army. The death-rate, which in February 1855 was 42 per cent., began to fall, and by June of that year it was only a little over 2 per cent.

After the Crimean War Miss Nightingale tried to work in peace so that never again should it be said that incompetence was responsible for the loss of life and the infliction of misery on our soldiers, whom she was in the habit of speaking of as her children. We were now reaping the reward of those efforts in the fine physique, staying-power, and general fitness of our troops to-day. In 1857 the rate of mortality in the Army in peace time was double that of the civilian population; for instance, the civil death-rate in the parish of St. Pancras was 2.2, but in the Life Guards Barracks 10.4; the civil death-rate in Kensington was 3.3, but in the Knightsbridge Barracks it was 17.5. This was chiefly due to the grossly insanitary and overcrowded state of the barracks, especially in regard to sleeping accommodation, for not infrequently each bed held four men. Many of the recommendations of Sidney Herbert were not accepted by the Commission, but some had since been adopted, and his work as a whole had formed the basis of manifold reforms. The health of our troops in India was notoriously bad, and Florence Nightingale said that 60 per 1,000 of our troops there perished. The annual cost of preventable disease in the troops was £388,000. The mortality in India was thought to be due to climatic conditions, and therefore inevitable. But the present death-rate among our troops in India was only 5 per 1,000. At one time it was the custom to bleed the men on the voyage to India, with the idea that the fresh blood made to replace that drawn off "would be more germane to the climate." Inasmuch as preventive medicine was unknown to the medical officers with armies at that date, they could not give sound advice for the health of the troops.

The Army Medical School Founded.

The first Army Medical School was founded at Chatham in 1860, and later it was removed to Netley. Its first Professor was Dr. Edwin Parkes, and the lecturer said it was impossible to overestimate the services which that excellent teacher rendered. He had seen service in India, and had written brilliant monographs on dysentery and cholera in that country, and in 1864 appeared the first edition of his work, "A Manual of Practical Hygiene," which served for more than a generation as a basis for the study of the principles of hygiene, and their application to military and civil life. At the time this appeared, our soldiers were clothed in a way most calculated to hinder their operations. The addition of heavy equipment (55 lbs.) caused wonder that he was able to accomplish as much as he did, especially owing to the very stiff stand-up collar which pressed under his chin. Though supplied with thin-soled and fairly tight boots, there were some wonderful marches to the credit of the troops at that date, a number of which he detailed.

With regard to the effects of alcohol, intemperance in the Army was now rapidly decreasing, as was fully borne out by statistics and by the enhanced good health of the soldier as a whole. Fifty years ago the British Army had the notoriety of being the most intemperate in Europe, and that was due to the stupid custom of serving out raw spirits as a regular ration. This did not quench but rather aggravated thirst, and any momentary exhilaration was followed by a depression which ill fitted the soldier for his arduous work. In 1864 Sir Hugh Rose reduced the spirit ration to half. The men who, in the American Civil War, were not paid owing to lack of funds, and therefore could not buy rum, were far healthier and more cheerful than the others, and were able to accomplish more. The wisdom of the Czar recently in prohibiting the sale of vodka throughout his dominions in the present crisis was borne out by results, and Dr. Sandwith said he sometimes wished that the magistrates of London and some other cities had been given greater powers in this regard. Still, alcohol administered in medicinal quantities at the end of a hard day's march proved a valuable stimulant for food when otherwise the men felt too tired to eat with relish, and men who normally were abstainers had found this produced an appetite. Alcohol was partly, though not wholly, responsible for the devilish performances of German soldiers and officers with unprotected women. The lecturer concluded by paying a glowing tribute to the organisation, foresight, training, and spirit of the men, as exemplified in the heroism of Dr. Rankin, who, after he had had his own thigh and leg shattered, still dragged himself about to attend to the wounds of others.

A number of interesting slides illustrating early conditions were shown at the close, the lecturer pointing out that at one time professional "bug-catchers" were appointed in hospitals, and they received the same salary as the surgeons!

